# Mobile medical simulation for rural anesthesia providers: A feasibility study

**DOI:** 10.36834/cmej.69572

**Published:** 2020-12-07

**Authors:** Kalyani Premkumar, Valerie Umaefulam, Jennifer M. O’Brien

**Affiliations:** 1 Department of Community Health and Epidemiology, College of Medicine, University of Saskatchewan, Saskatchewan, Canada; 2 Department of Anesthesiology, Perioperative Medicine and Pain Management, College of Medicine, University of Saskatchewan, Saskatchewan, Canada

## Abstract

**Introduction:**

Family practice anesthesia (FPA) providers are family physicians trained to deliver anesthesia care; they often practice in rural hospitals to facilitate surgical care. FPA providers in rural hospitals face challenges including professional isolation and limited opportunities for formal continuing education. To address needs identified by FPA providers, we piloted mobile medical simulation in rural Saskatchewan.

**Methods:**

Using a logic model framework, we evaluated feasibility of a one-day interdisciplinary mobile simulation workshop for healthcare providers in a rural Saskatchewan hospital. As part of this mixed methods pilot study, we interviewed stakeholders to explore their perceptions of human and financial resources associated with delivering medical simulations in rural locations. Multiple simulation scenarios were utilized to train participants in clinical and professional skills. Participants completed pre- and post-workshop surveys to evaluate their experience.

**Results:**

Financial and human resources included cost of renting, transportation of mannequins, and the time required to create the scenarios. Participants (*n* = 10) reported improved knowledge and found the experience valuable. The session prompted participants to reflect on their deficiencies in certain clinical procedures/skills and highlight learning strategies to address the gap.

**Discussion:**

Mobile medical simulation brought continuing medical education (CME) to health professionals in a rural location, but the program was expensive. Our logic model may inform educators and administrators considering mobile medical simulation for physicians in rural areas when balancing resource allocation and the organization’s commitment to CME for rural physicians.

## Abstract (fr)

**Introduction:**

Les anesthésistes en pratique familiale (APF) sont des médecins de famille formés pour offrir des soins d’anesthésie. Ils pratiquent souvent dans des hôpitaux ruraux pour faciliter les soins chirurgicaux. Les APF dans les hôpitaux ruraux sont confrontés à divers défis, tels l’isolement professionnel et un nombre limité d’occasions de formation continue formelle. Pour répondre aux besoins identifiés par les APF, nous avons mené une simulation médicale mobile dans une région rurale de la Saskatchewan.

**Méthodes:**

En utilisant un cadre de modèle logique, nous avons évalué la faisabilité d’un atelier sur une simulation mobile interdisciplinaire d’un jour pour les professionnels de la santé dans un hôpital rural de la Saskatchewan. Dans le cadre de cette étude pilote basée sur des méthodes mixtes, nous avons interviewé les parties prenantes pour explorer leurs perceptions en matière de ressources humaines et financières associées à la prestation de simulations médicales dans des zones rurales. Nous avons eu recours à de multiples scénarios de simulation pour former les participants dans des compétences cliniques et professionnelles. Les participants ont rempli un questionnaire avant et après l’atelier pour évaluer leur expérience.

**Résultats:**

Les ressources financières et humaines comprenaient le coût de location, le transport de mannequins et le temps requis pour créer les scénarios. Les participants (*n* = 10) ont rapporté avoir amélioré leurs connaissances et que l’expérience en valait la peine. La séance incitait les participants à réfléchir sur leurs lacunes dans certaines procédures ou compétences cliniques et à préciser des stratégies d’apprentissage pour combler leurs lacunes.

**Discussion:**

La simulation médicale mobile rapproche l’éducation médicale continue des professionnels de la santé dans une zone rurale, mais le programme a coûté cher. Notre modèle logique peut informer les éducateurs et les administrateurs qui envisagent la simulation médicale mobile pour les médecins dans les zones rurales lorsqu’ils équilibrent les ressources et l’engagement de l’organisation à la formation médicale continue pour les médecins en zones rurales.

## Introduction

Family Practice Anesthesia (FPA) providers are family physicians who have earned a certificate of added competence to deliver anesthesia care. FPAs provide the vast majority of anesthesia care in rural Canada, and are essential to the delivery of surgical and obstetric care in rural communities across the country.^1^ However, there is a shrinking number of rural FPAs due to factors including burnout, isolation, and lack of opportunities for professional development.^2^

Opportunities for continuing medical education (CME) for rural FPA providers are geographically distant, costly, and logistically challenging.^2^ Rural physicians find it challenging to maintain skills due to the lower rates of complex procedures in rural practice.^3^ In a recent survey of Canadian FPAs, respondents indicated that refresher time in a centre with a larger volume of complex cases and formal mentoring with specialist anesthesiologists or FPAs would support their continued practice.^4^ Innovative strategies and approaches are required to strengthen and stabilize anesthesia care teams working in rural regions.^1^ FPA providers have indicated the need for CME that supports sharing experiences and obtaining mutual support from colleagues.^2^

Medical simulation (MS) imitates an interprofessional team environment and is used to provide training to healthcare providers (HCPs) with an opportunity and safe space to learn, practice skills, and make mistakes without harm to real patients.^5^ Researchers investigating mobile simulation educational sessions for rural healthcare providers in nine southern Manitoban communities showed that although participants had limited or no previous access to high fidelity mannequins, they expressed high level of satisfaction with the simulation sessions with high ratings of the quality of learning, decision making proficiencies, and clinical reasoning skills.^3^ Inspired by mobile simulations in Manitoba, we sought to evaluate the feasibility and effectiveness of a mobile simulation intervention for improving self-reported clinical and professional skills in rural Saskatchewan healthcare providers.

## Methods

Using a logic model framework,^6^ we evaluated the feasibility of a one-day interprofessional mobile simulation workshop for 10 participants, including registered nurses and FPA providers. We used a logic model (Table 1) as a process tool during the program planning, implementation, and evaluation which helped us identify the various components (e.g. stakeholders, equipment, cost, etc.) and relationships between them. Following research ethics exemption as a program evaluation activity under Article 2.5 of the Tri-Council Policy Statement, we conducted a mixed methods pilot study. Primary outcomes included the financial and human resources required to run the workshop. Secondary outcomes included evaluation of the impact on participant knowledge and clinical skills using self-reported pre-post surveys and stakeholder interviews. Prior to survey distribution, we established face validity by having it reviewed by four anesthesiologists and educators and then revised the questions according to their feedback.

**Table 1:** A logic model for evaluation of mobile medical simulation in rural Saskatchewan

Objectives:			
1) To deliver continuing medical education opportunities for skill development to rural Family Practice Anesthesia providers and registered nurses; 2) To improve peer communication; and 3) To practice skills.			
**Inputs** *Resources invested*	**Activities** *Actions carried out*	**Outputs** *Products and services delivered*	**Outcomes** *Expected changes*
**Money**: $6,000 for high-fidelity mannequin rental and computer software from private sector; transportation costs reimbursed as per university policy; physician facilitators’ payment **Human resources**: two simulation-trained anesthesiologist workshop facilitators, including time to edit existing scenarios (2-3 hours per scenario; 8-12 hours total) and deliver the 1-day workshop **Facilities**: Rural hospital 175 km north-east of Saskatoon **Equipment**: High-fidelity mannequin and computer software, task-trainers, video recording equipment	Edit scenarios previously developed for anesthesiology resident training Travel to rural community hospital for simulation set up	Deliver 4 medical simulation scenarios in a rural community hospital: Anaphylaxis Intraoperative arrest Can't Intubate, Can't Ventilate Medicolegal Facilitators conduct extensive debrief discussion session to help participants reflect on the scenarios	*Expected Short Term Outcomes* Direct result of outputs Opportunity for interprofessional training and building relationships between rural and urban physicians High quality learning in a safe environment Comfort in managing crisis Identify system issues that they may not have known (e.g. equipment, drug, more hands) to manage a difficult situation *Expected Intermediate Outcomes* Changes anticipated after achievement of immediate outcomes Improved clinical and professional skills Enthusiasm for mobile simulation *Expected Long Term Outcomes* Furthest reaching changes attributable to the program Build a provincial network of simulation Success of mobile simulation as a training tool in rural healthcare
**Challenges** Lack of technical support for simulation equipment, time required to set up mannequins, and various cost issues (including transport of physicians & equipment, mannequin rental from a private sector business, physician facilitators’ payment, credit application).			

### Stakeholder participant characteristics

Prior to the workshop, we conducted semi-structured face-to-face or telephone interviews with key stakeholders to identify the workshop objectives. Stakeholder participants who informed the workshop objectives and content included a senior leader in the Department of Anesthesiology, three urban specialist anesthesiologists who were also simulation specialists; two workshop facilitators; eight potential participants of the workshop, and leaders/administrators in the rural location. Themes from interviews with key stakeholders informed workshop content.

### Intervention

Two specialist anesthesiologists trained in providing medical simulation facilitated the one-day workshop in rural Saskatchewan. They delivered four scenarios (previously used for training anesthesiology residents) that were comprehensive and addressed important clinical skills required in anaesthesiology and for delivery of care in a rural environment. The scenarios covered the following topics: Anaphylaxis, Intraoperative arrest, Can't Intubate Can't Ventilate, and Medicolegal issues. High fidelity mannequins attached to computer software were chosen for their ability to manifest physiological changes based on the clinical interventions administered by participants. Task-trainers were available to help participants practice specific procedures such as central line and chest-tube insertion. Following each simulation scenario, facilitators led a comprehensive debriefing for participants using selected illustrative video recordings of the sessions to provide meaningful feedback about changes that could be made.

### Data collection

We conducted semi-structured interviews with facilitators post-workshop to explore their perceptions of human and financial resources required to run the mobile simulation scenarios in rural Saskatchewan.

Since learner reactions and perceptions to the simulation experience are often used for evaluation,^7^ all 10 participants completed a pre-workshop survey (Appendix A) rating the extent to which they were currently able to meet the workshop objectives on a 5-point Likert scale, where 1 = Not at all, and 5 = Very much. Immediately after the workshop, participants rated their self-reported perception of the workshop’s impact on clinical and professional skills, and learning gained from the case scenario sessions. Three participants were randomly selected to complete the 7-point Debriefing Assessment for Simulation in Healthcare (DASH)^©^^8^ for each scenario, to collect feedback for future iterations of the workshop. One month following the workshop, we sought participant feedback to evaluate workshop content, and seek participant perceptions of whether and how the workshop changed their clinical practice. We deemed one month to be an adequate period for participants to reflect on the experience and practice skills learned.

### Data analysis

Qualitative data were analyzed using NVivo 12. The thematic analysis involved in-vivo coding of responses, development of themes from the coded interviews, and refinement and description of themes. Survey data were analyzed using SPSS to provide descriptive analysis and to compare pre-post change in survey scores. All responses to open-ended questions were de-identified and coded for common themes.

## Results

Pre-workshop, stakeholders anticipated barriers to mobile medical simulation that included human and financial resources associated with infrastructure, time and travel logistics, the perceived interest in MS by HCPs, and the ability to implement MS CME sessions. Themes and subthemes from interviews with key stakeholders are presented in Appendix B, with illustrative quotations.

### Workshop participant characteristics

Ten healthcare professionals (nurses = 5; physicians = 5) participated in the workshop. Participants were predominately female (80%), aged between 30 and 50 years (67%), and reported 1-15 years of post-training work experience.

### Post-workshop facilitator interviews

Workshop facilitators identified challenges such as human and financial resources required to implement medical simulation in a rural community, including lack of technical support, the time required to set up mannequins, and various cost issues (such as transport of physicians & equipment, mannequin rental from a private sector business, physician facilitators’ payment, application for Continuing Professional Development (CPD) credits, among others); they felt that these challenges negatively impacted the implementation of the workshop. However, facilitators further commented that the workshop created the opportunity for interprofessional training and building relationships between rural and urban physicians.

### Pre and post-workshop survey results

Mean self-reported clinical and professional skills scores improved, and standard deviations narrowed in each category between pre- and post-surveys. The total mean score of survey responses pre-workshop was 3.22 (SD=1.10) and post-workshop, the mean score was 3.71 (SD=0.88, effect size 0.49). The pre-post change in scores was not significant. Participants reported that the workshop was impactful in enhancing communication with peers, patients, and family members, notably in survey questions pertaining to communication on medio-legal issues and disclosing health information to a patient or family. Despite reminders sent via email to participants, only one participant completed the one-month follow-up survey, reporting better ability to manage bronchospasm, anesthetic toxicity, hypoxia, anaphylaxis, and post anesthetic complications. In addition, the participant reported knowing how best to disclose an adverse event to a patient or family and was very interested to participate in continuing medical education and skill development.

### Debriefing scores

Debriefing scores showed that participants perceived the medico-legal scenario as extremely effective (mean = 7, Extremely Effective/Outstanding). Participants indicated that the scenarios on intraoperative arrest, can’t intubate can’t ventilate, and anaphylaxis were Consistently Effective/Very Good (mean = 6). Post-workshop, participants identified deficiencies in their clinical skills (e.g. epinephrine dosing for anaphylaxis; technique for cricothyrotomy) and learning strategies to address the gaps (e.g. will review vasopressin doses; will periodically review technique for cricothyrotomy).

## Discussion

We conducted a pilot study to evaluate the feasibility of conducting a mobile medical simulation workshop in rural Saskatchewan. While other jurisdictions have had some success in establishing robust rural simulation programs, our College of Medicine did not choose to support this initiative beyond the initial pilot, likely due to the resource-intense nature of renting the mannequin for the mobile workshop from a private sector simulation institute, reimbursing travel expenses, and payment for physician facilitator time. At the time, our College of Medicine mannequins could not be taken out of University facilities for rural simulation because this fell outside the University’s mandate. These challenges underscore the need for communicating the value and importance of stakeholder support to organizational leadership.^9^

The small number of participants, self-report of participants perceptions, and the low response rate for the 1-month post-workshop questionnaire limit generalizability due to selection bias and reporting bias. The low response rate underscores the constraints faced by rural healthcare practitioners such as long work hours, inadequate health workforce and resources, and heavy on-call responsibilities.^1^ However, the single respondent expressed an interest in further participating in medical simulation programs for CME and skills development, which is congruent with the high level of satisfaction expressed by participants in similar studies of mobile simulation in rural environments.^3^

The use of pre-existing scenarios developed for anesthesiology residents enabled the provision of this mobile simulation workshop. Facilitators were already familiar with these scenarios, which may have reduced the burden of facilitating the MS. However, creating and editing the scenarios still required about 2-3 hours per scenario (8-12 hours total). A strength of this workshop was the ability to provide customized scenarios for an interprofessional rural health team in a familiar and authentic location of practice, a benefit which has been demonstrated in similar studies.^3^^,^^10^ Training of rural physicians in facilitating simulations could reduce several limitations including unfamiliarity with the local set up.

Challenges to our pilot feasibility study of mobile MS included the facilitators’ unfamiliarity with the local set up, and the lack of availability of technology and resources in the rural setting. These challenges may have been mitigated through a site visit early in the planning stages.^9^ Although a technician was not part of the simulation team, the facilitators were able to carry out both the simulation and debriefing. Nevertheless, the facilitators experienced some technical difficulty during set up; we may have underestimated the technical needs of running the simulation. Therefore, having a technical team member may have made workshop set up more efficient.^9^ Transporting the mannequins to the rural location was costly, due to the great distances between urban and rural areas in Saskatchewan. Some Canadian centers have developed a dedicated mobile simulation unit with the academic mandate of providing medical simulation in rural and remote areas.^3^^,^^10^^,^^11^ Emerging interest in simulation delivered via video teleconference may address many of the challenges that arose due to equipment transport and facilitator time.^9^ Parsons et al. are investigating telesimulation, the use of virtual medicine technologies to deliver meaningful simulation experiences to learners in rural and remote locations.^11^ The potential to incorporate the use of telesimulation in rural settings could help reduce the need to pay for facilitator travel time.^11^

## Conclusion

Within a logic model framework, this report highlights the objectives, resources, and activities invested to deliver mobile medical simulation to rural HCPs in Saskatchewan. Mobile medical simulation brought continuing medical education to health professionals in a rural location, but the program was expensive. Our logic model may inform educators and administrators considering mobile medical simulation for physicians in rural areas when balancing resource allocation and the organization’s commitment to CME for rural physicians. Results suggest that – despite the resource-intensive nature of mobile simulation activities – it is valuable to make CME training sessions more accessible in rural settings and provide medical simulation in the same environment that services are delivered. Feedback from workshop participants suggests that mobile medical simulation has the potential to improve the clinical and professional skills of anesthesia providers in rural areas, but future studies with more participants are required. Future medical simulation workshops should consider delivering simulation via videoconference, establishing a travelling mannequin repository to reduce recurring costs, and using other methods of long-term evaluation to obtain richer feedback and objective measures of competencies.

## Appendix A. Pre-workshop survey of participants

Name:_________________________________

Gender: M F (please circle)

Age: (please circle) <30; 30-40; 40+-50; 50+-60; >60

1. In which Saskatchewan health region do you currently practice?
  Athabasca
  Cypress
  Five Hills
  Heartland
  Keewatin Yatthe
  Kelsey Trial
  Mamawetan Churchill River
  Prairie North
  Prince Albert Parkland
  Regina Qu’appelle
  Saskatoon
  Sun Country
  Sunrise
2. Where did you complete your training program in family medicine?
3. Where did you complete (or are completing) your training program in anesthesia?
4. How many years have elapsed since you completed your residency? (Please check the appropriate response below)
  Not completed 1-2 3-5 6-10 11-15 16-20 21-25 26-30 Over 30
5. Please indicate what you expect to achieve in today’s workshop?
6. The questions below reflect the Goals and Objectives of the training requirements in Family Practice Anesthesia (FTA)in Saskatoon. For each item, please rate the extent to which you think you are able to meet that objective. As well, please indicate whether you would like to have the option to have an educational program to supplement your knowledge and skills in that area.

**Table:**

To what extent …	Not at all				Very Much
… are you able to identify cases that may be beyond the capabilities of either the anesthetist or facility?	1	2	3	4	5
… are you able to recognize which patients require immediate stabilization and transport to a tertiary care facility?	1	2	3	4	5
… are you able to perform advanced and ancillary techniques for intubation?	1	2	3	4	5
… use anesthesia machine and demonstrate an understanding of its principles and basic maintenance?	1	2	3	4	5
… are you able to perform acute resuscitation during cardiac arrest?	1	2	3	4	5
… respond to the special needs of ambulatory patients?	1	2	3	4	5
… respond to urgent anesthesia (when the safety of the patient might be compromised during transportation)?	1	2	3	4	5
… respond to elective anesthesia (to maintain surgical/anesthetic support skills for the convenience of the patient and community)?	1	2	3	4	5
**During trauma management, to what extent are you able to …**	1	2	3	4	5
… perform cardio-respiratory stabilization?	1	2	3	4	5
… insert vascular lines?	1	2	3	4	5
… assess the status of a patient?	1	2	3	4	5
… evaluate the urgency of surgery?	1	2	3	4	5
… appropriately manage acute or chronic cardiac arrhythmias or myocardial infarction?	1	2	3	4	5
… participate in continuing medical education and skill development?	1	2	3	4	5
… communicate with other members of the healthcare team to benefit the patient?	1	2	3	4	5
… know the CAS (Canadian Anesthesiologists’ Society) guidelines for management of patients in the perioperative period?	1	2	3	4	5
… regularly review procedures/policies with the goal of detecting areas of potential improvement?	1	2	3	4	5
Intraoperative hypotension	1	2	3	4	5
… critically evaluate the medical literature pertaining to anesthesiology as it applies to FPA practice?	1	2	3	4	5
Intraoperative bronchospasm	1	2	3	4	5
Unable to ventilate or intubate	1	2	3	4	5
Intraoperative anaphylaxis	1	2	3	4	5
intraoperative hypoxia	1	2	3	4	5
Intraoperative equipment failure with inability to positive pressure ventilate by bag or ventilator	1	2	3	4	5
intraoperative cardiac arrest	1	2	3	4	5
tension pneumothorax	1	2	3	4	5
aspiration pneumonitis	1	2	3	4	5
local anesthetic toxicity	1	2	3	4	5
post anesthesia problems of hypoxia	1	2	3	4	5
post anesthesia problems of hypotension	1	2	3	4	5
post anesthesia problems of dyspnea	1	2	3	4	5
post anesthesia problems of agitation	1	2	3	4	5
post anesthesia problems of narcotic overdose	1	2	3	4	5
post anesthesia problems of narcotic reversal	1	2	3	4	5
post anesthesia problems of obstetrical bleeding	1	2	3	4	5
Total spinal	1	2	3	4	5
How to assess the volume status of a patient who must be anesthetized emergently	1	2	3	4	5
How to diagnose and treat pulmonary hypertension intraoperatively	1	2	3	4	5
How to diagnose and treat anaphylaxis intraoperatively	1	2	3	4	5
When to call a code in the operating room	1	2	3	4	5
Understand the physiological changes during spinal anesthesia that might produce a cardiac arrest	1	2	3	4	5
how to differentiate between bronchospasm from asthma and bronchospasm from anaphylaxis	1	2	3	4	5
Awareness of coronary insufficiency and anaphylaxis	1	2	3	4	5
Determine how to assist colleague in can’t intubate scenario	1	2	3	4	5
Determine indications for a surgical airway	1	2	3	4	5
Determine the events that might lead to a cardiac arrest in the operating room	1	2	3	4	5
Determine ways in which poor communication and team skills can affect patient outcome	1	2	3	4	5
How best to disclose an adverse event to a patient or family	1	2	3	4	5
Understand the medical-legal process of being named in a medical law suit	1	2	3	4	5
Understand the personal mal-adaptive changes that occur as a second victim	1	2	3	4	5
Understand ways that will assist a physician dealing with medical-legal law suit	1	2	3	4	5

Are you required to manage invasive monitorings?

Yes No

If Yes, how well are you able to manage this?

Not at allVery Much

12345

**The following questions relate to Stimulations**.

Have you had any prior experience with medical stimulation?

Yes No

If yes, please describe:

Please indicate which areas of stimulation that would be useful for you. Select as many as you like.

Task trainers for central line

Chest tube insertion

Pericardial centesis

Failed intubation

Surgical airway

Disclosure

Breaking bad news

Impaired physicians

Resuscitation of pregnant patient

Cervical spine injury

Acute renal failure

Burns

Stroke

Special problems associated with air evacuation

Others: please specify

Please provide any comments you have about the needs of Family Physician Anesthesiologists in rural areas:

***Thank you for completing this survey. Your Feedback is greatly appreciated!***

## Appendix B. Stakeholder perceptions (themes and sub-themes)

**Table:**

Theme 1: Objectives of the workshop	
***Subthemes***	***Illustrative quotations***
CME and skills development	“ongoing maintenance of CANMEDS milestones/ continuing medical education” “assist in the training of family physicians to manage rare but potentially life-threatening situations such as ACLS, PALS, drug and toxin overdose, and trauma” “non-technical skills such as communication and team skills”
Peer communication	“chance to link with community anesthesia practitioners” “To improve communication among health care team members” “to see an improvement in the ability of the rural docs in identifying mentors in Saskatoon, should they require assistance or advice in their day-to-day activities in their own operating rooms.”
Practice of skills	“*safe environment to practice skills that are not practiced on a regular basis” “give rural GPs exposure to management situations that they may not see frequently enough in practice” “assess and enhance existing skillsets” “to hone skills in acute treatment”* and *“practice of leadership skills in crisis situations”*
**Theme 2: Predicted short term outcomes**	
***Subthemes***	***Illustrative quotations***
Enthusiasm for MS	“enthusiasm for more simulation opportunities by participants” “excitement and enthusiasm from those involved”
High quality learning in a safe environment	“Forum for discussion of techniques and troubleshooting” “increase knowledge about the cases/scenarios” “present an opportunity to deal with rare situations in a non-threatening environment, refreshes things and puts them in perspective” “As an operative nurse in OR: Information regarding care occurrences and complications that could occur during operative procedures to be better equipped to assist anesthetist that could improve patient outcome”
Managing crisis	“Comfort in managing a crisis with a safe place to practice” “Identify system issues that they may not have known (e.g. equipment, drug, more hands) to manage a difficult situation” “brush up on emergency situations in a controlled environment”
**Theme 3: Predicted long term outcomes**	
***Subthemes***	***Illustrative quotations***
Build a network of simulation	“this will be the start to building a provincial network of simulation” “create a network for sustaining FPA practice in Saskatchewan”
Success of MS	“The rural healthcare community would need to find this a very effective training tool. ‘Just okay’ isn’t good enough; it’s got to be spectacular.”
Comfort with skills	“expand the scope of their capabilities, comfort level”
Education event and CME	*“independent practitioners will want to continue as part of CME (increase demand for simulation opportunities)”* CME *“may become an ongoing project… go from region to region and deliver educational event” “more involvement of family physicians in CME activities, perhaps the expansion of workshops such as this so family physicians could get more education without leaving their home, and perhaps less feelings of personal isolation”*
Improve healthcare and patient safety in the community and province	“Simulation may form a key part of quality improvement (health systems and patient outcomes) by being able to reproduce the incident and more carefully analyze it.” “It’s critical for Quality Control, and for urban/rural people to get to know each other.”
Support rural centres	“Might prompt continued investment by the Ministry of Health in innovations to support rural centers” “development structure simulation program for rural practitioners – anesthesia, trauma management, critical care mgt, acute med emergency mgt”
**Theme 4: Utility of Mobile Simulation in continuing professional education**	
***Subthemes***	***Illustrative quotations***
New way of learning	“The scope of what can be offered is quite varied and can be tailored to the end-user” “New modality better than didactic. Immersive experience.”
Patient safety	“Hopefully improve patient safety and risk as trainees are learning.” “It is not only feasible, but absolutely necessary, particularly with number of preventable medical errors. This will increase patient safety.”
**Theme 5: Challenges**	
***Subthemes***	***Illustrative quotations***
Infrastructure for high quality simulation	“The College of Medicine has decreed that its simulation equipment can’t leave the university. To enable us to bring simulation equipment to the rural areas – we learned of the new simulation center in Saskatoon from which we can draw equipment and expertise to do these things” “Physical location, equipment, etc., may make it difficult to recreate the environment.”
Human Resources	*“Stress on the team from Saskatoon to go. They leave early and come home late. Will they come home exhausted and never want to do it again?” “developing a dedicated faculty, and mobile service with support” “Having a mannequin operator with anesthesia background, so they know what the critical things are, and can give applicable answers to questions” “weather in SK (for travel)”*.
Participants’ Interest	“Participants need to be interested in this program.” “Are people going to show up?” “Receptivity of FPAs to engage” “getting everybody together in the same time & place. Pulling a group of doctors and nurses out of a community at the same time limits the access to services in that community. Must be creative.”
Resources and Money	“FINANCIAL - because goodwill only goes so far, we have sponsorship from Sask Medical Assn. for this time – this one on Saturday.” “Depends on ongoing funding. Simulation is an expensive way of educating” “Needs leadership and visionaries for structure and sustainability”. “SIMS can provide this service for the province at a more cost-effective way than every health region developing their own sim center.”
